# Epidemiology, clinical presentation, and predictors of outcome in nontuberculous mycobacterial central nervous system infection: a systematic review

**DOI:** 10.1186/s41182-023-00546-4

**Published:** 2023-09-25

**Authors:** Durga Shankar Meena, Deepak Kumar, Vasudha Meena, Gopal Krishana Bohra, Vibhor Tak, Mahendra Kumar Garg

**Affiliations:** 1 Department of Internal Medicine (Division of Infectious Diseases), All India Institute of Medical Sciences, Jodhpur, 342005 India; 2 Department of Pediatrics, Dr. S.N. Medical College, Jodhpur, 342005 India; 3 Department of Microbiology, All India Institute of Medical Sciences, Jodhpur, 342005 India

**Keywords:** Nontuberculous mycobacteria, NTM, Central nervous system, Brain abscess, Meningitis

## Abstract

**Background:**

CNS manifestations represent an emerging facet of NTM infection with significant mortality. Due to protean presentation and low index of suspicion, many cases are often treated erroneously as tubercular meningitis or fungal infections.

**Objectives:**

Literature on NTM CNS disease is scarce, with most available data on pulmonary disease. This systematic review aimed to evaluate the epidemiology, clinical presentation, diagnostic modalities, and predictors of outcome in CNS NTM infection.

**Methods:**

The literature search was performed in major electronic databases (PubMed, Google Scholar, and Scopus) using keywords “CNS,” “Central nervous system,” “brain abscess,” “meningitis,” “spinal,” “Nontuberculous mycobacteria,” “NTM”. All cases of CNS NTM infection reported between January 1980 and December 2022 were included.

**Results:**

A total of 77 studies (112 cases) were included in the final analysis. The mean age of all patients was 38 years, with most patients male (62.5%). *Mycobacterium avium complex* (MAC) was the most common aetiology, followed by *M. fortuitum* and *M. abscessus* (34.8%, 21.4% and 15.2%, respectively). The disseminated disease was found in 33% of cases. HIV (33.9%) and neurosurgical hardware (22.3%) were the common risk factors. Intracranial abscess (36.6%) and leptomeningeal enhancement (28%) were the most prevalent findings in neuroimaging. The overall case fatality rate was 37.5%. On multivariate analysis, male gender (adjusted OR 2.4, 95% CI 1.2–7.9) and HIV (adjusted OR 3.7, 95% CI 1.8–6.1) were the independent predictors of mortality). *M. fortuitum* infection was significantly associated with increased survival (adjusted OR 0.18, 95% CI (0.08–0.45), *p* value 0.012).

**Conclusions:**

Current evidence shows the emerging role of rapid-grower NTM in CNS disease. Male gender and HIV positivity were associated with significant mortality, while *M fortuitum* carries favourable outcomes.

**Supplementary Information:**

The online version contains supplementary material available at 10.1186/s41182-023-00546-4.

## Introduction

Nontuberculous mycobacteria (NTM) are ubiquitous free-living organisms in the environment. Since the advent of modern microbiological methods, the importance of NTM infections is increasingly evident. Phenotypic methods are helpful in resource-limited settings. However, long turnaround time is the major limitation. This genus has more than 170 species, and the numbers are growing rapidly with the recent advancement in genotypic diagnostics (16S rRNA gene sequencing, metagenomics) [1, 2]. Mycobacterial 16S rRNA is a highly preserved nucleotide sequence; a 1% or more difference in this sequence can be labelled as a new NTM species [1, 3]. Short turnaround time and quick availability of drug susceptibility are the main advantages of genotypic methods. Traditionally considered an opportunistic infection (associated with HIV), NTM infections are increasingly recognized in immunocompetent patients due to improved diagnostic methods and other predisposing conditions, such as old pulmonary tuberculosis, cystic fibrosis and chronic obstructive pulmonary disease. The prevalence of NTM among HIV patients ranged from 1.3% to 27.3% compared to tuberculosis (ranging from 11.8% to 48.7%) [4]. Disruption of the IFN-γ/IL-12 axis in HIV patients is the main trigger for disseminated NTM infections. Toll-like receptors (TLRs) on macrophages sense mycobacteria and release cytokines (IL-12 and TNF-α); this stimulates IFN-γ production and Th1 cell differentiation in HIV patients and makes them vulnerable to NTM infections [5]. Compared to HIV, the incidence of NTM disease in the population is 3.1 to 4.7 per 100,000 person-years [6]. CNS infections by NTM are rarely described in the literature, with most cases associated with HIV and MAC infections [7]; however, rapidly growing mycobacteria are now getting more attention as an aetiology of CNS NTM disease [8]. Meningitis is the most commonly reported presentation in the literature, with a high mortality rate (up 77%) [7].

CNS infections associated with NTM are challenging to diagnose, with many patients erroneously treated with conventional antitubercular drugs. These drugs are partially effective in NTM disease, resulting in treatment failure with high morbidity and mortality [9]. Efflux pumps, porin channels, and polymorphism in the target genes are important mechanisms for intrinsic resistance of NTM for antitubercular drugs [9]. We conducted a systematic review of all reported cases with CNS NTM disease to gain new insight into these patients' clinical characteristics, aetiology, risk factors, treatment, and prognosis. There are no guidelines for CNS NTM infections, with most literature focused on pulmonary disease. We chose to select CNS cases for review, which have higher mortality and closely resembles tubercular and fungal CNS infections, which makes the diagnosis challenging. Our object was to generate evidence from the available literature that can contribute to subsequent studies and formulate treatment protocols for these patients. Moreover, it will help to aware clinicians of this uncommon presentation.

## Methods

### Protocol and registration

This systematic review is performed in accordance with the Preferred Reporting Items for Systematic Reviews and Meta-Analyses (PRISMA) statement (Additional file 1) [10] and is registered in the PROSPERO online database (PROSPERO Identifier: CRD42023405524).

### Search strategy and information sources

We performed a systematic search of the literature to identify all the reported cases of CNS disease due to nontuberculous mycobacteria. The literature search was performed using different electronic databases of the English literature (PubMed/Medline, Google Scholar and Scopus). A manual search was also performed to identify the cases. The case reports and case series published between 1980 and 2022 were identified and retrieved. The literature published before 1980 was difficult to retrieve and lacked complete clinical information, henceforth excluded. The search terms (keywords) for this systematic review were: “CNS,” “Central nervous system,” “brain abscess,” “meningitis,” “spinal,” “nontuberculous mycobacteria” and “NTM” in different combinations (Additional file 1). Meningitis and brain abscess were the commonest CNS manifestations; hence these search terms were included.

### Study selection (case definition and inclusion criteria)

We included 112 cases of CNS nontuberculous mycobacterial infection in this study. The cases fulfilling the following criteria were included in the final analysis: a). Cases with isolation of NTM from the culture of a sterile site (CSF, abscess, or tissue sample), compatible with the clinical–radiological syndrome (CNS disease) or b). Identification of NTM species by molecular methods (e.g., PCR or next-generation sequencing) with a compatible clinical–radiological syndrome. Disseminated infections with isolation of NTM from pulmonary samples were also included. Reports with culture-positive NTM without evidence of clinical CNS disease were excluded. Both pediatric and adult cases were included. Data regarding clinical presentation, diagnostic modalities, treatment and outcomes were documented. Treatment of NTM infections is usually prolonged (around 12 months after culture conversion, depending on the patient's immune status); thus, only cases with a follow-up of a minimum of 2 months were analyzed for the outcome. It takes several weeks to achieve antibiotics response in NTM; a 2-month follow-up was chosen after the consensus with all the reviewers. Abstracts, editorials, review articles, conference papers and posters were excluded. Cases with solid organ or stem cell transplant recipients, patients on corticosteroids (≥ 10 mg/day for at least 4 weeks) or treated with long-term immunosuppressants were defined as immunocompromised. Disseminated NTM disease was defined as a case of CNS disease along with the evidence of NTM isolation from blood (NTM bacteremia) or involvement of 2 or more non-contiguous organs such as CNS with involvement of the lung, bone marrow, abdomen or lymph nodes.

### Data extraction and qualitative assessment

Two investigators (DSM and DK) independently extracted the clinical details from the selected cases. The online software for systematic review (Covidence systematic review software, Veritas Health Innovation, Melbourne, Australia) was used to extract the data. The following data were extracted: clinical presentation, presence of risk factors, diagnostic procedures, antibiotic therapy and outcome with follow-up. The disparity between the authors was resolved by the discussion and consensus with the other reviewer authors (VM, VT, GKB). Case reports are associated with inherent bias; to decrease this, we adopted the standardized critical appraisal tool proposed by the Joanna Briggs Institute (JBI) [11]. Each study was assessed based on the availability of data (clinical presentation, diagnosis, treatment, adverse events, and takeaway message) and rated with the probability of bias (Low risk = ≥ 7 yes; Moderate risk = 5–6 yes; High risk = ≤ 4 yes). We have attached the JBI critical appraisal checklist in the Additional file 1.

### Statistical analysis

The SPSS software version 20.0 (IBM Corp, Armonk, NY) was used for data analysis. Descriptive data were compiled and tabulated with continuous variables in the form of mean ± standard deviation, median (with interquartile range), and categorical variables in the form of a number (percentages). Pearson’s Chi-square test was used to analyse the difference between categorical variables. Univariate regression analysis was used to identify the predictors of mortality. The variables with significant correlation (*p* value ≤ 0.05) were further selected for multivariable regression analysis. The results were expressed as an odds ratio with a relative 95% confidence interval and *p* value. A *p* value < 0.05 was considered to indicate statistical significance.

## Results

**Fig. 1:**
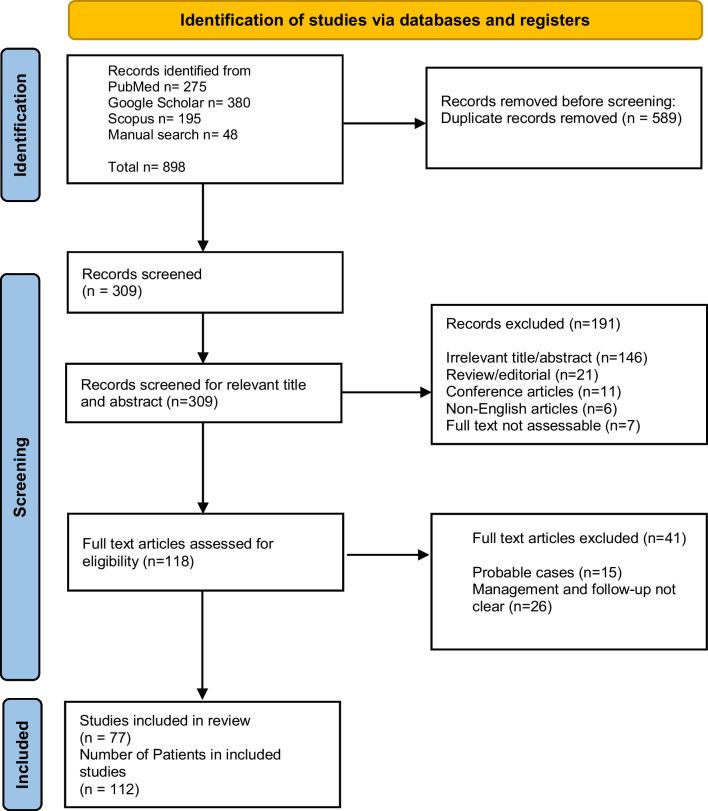
Flow chart of articles selection according to PRISMA guideline

A total of 898 case records were identified from the initial literature search. Three hundred nine studies were further assessed after excluding duplicate case records. After removing ineligible cases, 112 patients (77 studies) with CNS NTM infection were included and analysed in this review (Fig. 1). 71 records out of 112 were individual data, and the remaining 41 patients were from case series (8 studies). The references of all the cases included in this systematic review are provided in the Additional file 1.

### Patients’ demographic characteristics

The mean age of the patients was 38.2 ± 17.8 years (Table 1). Sixteen patients (14.3%) were from the pediatric age group (< 18 years), while 96 patients (85.7%) were adults. The proportion of male patients was 62.5%. Most cases of CNS NTM disease were reported from the USA (50.9%), followed by Asia (34.8%) and Europe (12.5%). Notwithstanding, it may not be an accurate representation due to the large population of South Asia and the high burden of pulmonary NTM infections. The fewer cases from this region indicate a lack of diagnostics and awareness about CNS manifestations of NTM infection.

**Table 1:** Clinical and demographic characteristics of patients with CNS NTM infection

Characteristics	*N* = 112
Age range (years) (*n* = 112)	1.5–82 years
Pediatric cases, age range (years) (*n* = 16, 14.3%)	1.5–17 years
Adult cases age, range (years) (*n* = 96, 85.7%)	18–82 years
Overall Mean age ± SD	38.2 ± 17.8 years
Overall Median age	36 years
Gender (*N* = 112)	
Male	70 (62.5%)
Female	42 (37.5%)
Geographical distribution (*N* = 112)	
USA	57 (50.9%)
Asia	39 (34.8%)
Europe	14 (12.5%)
Australia	1 (0.9%)
Africa	1 (0.9%)
Case fatality	42/112 (37.5%)
Disseminated NTM disease	37/112 (33%)
Immunocompromised	51/112 (45.5%)

### Etiology and epidemiological trend

We have categorized all CNS NTM cases based on the type of NTM species isolated from CSF or other sterile tissue, which is described in Table 2. Rapid-growing NTM (RGM) were isolated from 54.5% of the cases (61 cases), and slow-growing NTM (SGM) from 45.5% (*n* = 51) cases. Overall, MAC was the most common cause of CNS infections (34.8%, *n* = 39), followed by *M. fortuitum* (21.4%, *n* = 24) and *M. abscessus* (15.2%, *n* = 17). Notwithstanding, *M. fortuitum* was the most common aetiology of NTM CNS disease (75%) in the pediatric population. We have also analyzed the epidemiological trend of NTM CNS disease over the past 42 years (1980 to 2022); MAC was the commonest isolate in cases reported from 1980 to 2000 (69.23%, 18 cases out of 26), though it was found in only 24.4% (21 out of 86) of the cases reported from 2000 to 2022. Conversely, the RGM were the major isolate in the past 20 years (65%) responsible for CNS infections.

**Table 2:** Distribution of various NTM species causing CNS manifestations

Type of nontuberculous mycobacteria/species	Total number of patients (*N* = 112)
Rapid-growing nontuberculous mycobacteria (RGM)	61 (54.5%)
Slow-growing nontuberculous mycobacteria (SGM)	51 (45.5%)
*M. avium *complex (SGM)	39 (34.8%)
*M. fortuitum* (RGM)	24 (21.4%)
*M. abscessus* (RGM)	17 (15.2%)
*M. haemophilum* (SGM)	7 (6.6%)
*M*. *massiliense* (RGM)	6 (5.4%)
*M. kansasii* (SGM)	4 (3.6%)
*M. mucogenicum* (RGM)	4 (3.6%)
*M. chelonae* (RGM)	4 (3.6%)
*M. goodii* (RGM)	3 (2.7%)
*M. bolletii* (RGM)	1 (0.9%)
*M. simiae*^***a***^ (SGM)	1 (0.9%)
*M. immunogenum*^b^ (RGM)	1 (0.9%)
*M. houstonense* (RGM)	1 (0.9%)

a = 40-year-old diabetic male with mixed infection of *Mycobacterium simiae* and *Mycobacterium Haemophilum* (both species identified by 16 s ribosomal RNA gene sequencing), b = polymicrobial infection in a brain abscess patient, including 2 rapidly growing NTM species (*Mycobacterium immunogenum* and *Mycobacterium llatzerense*)

### Clinical features and risk factors

Fever was the most predominant presenting symptom, followed by headache and altered mental state (58.9%, 35.7% and 29.5%, respectively) (Table 3). Among neurological deficits, cranial nerve involvement was seen in 6 patients (6.9%). The abducens nerve was the most commonly involved nerve (4 out of 6 patients), with third, fourth, fifth and seventh other cranial nerves affected. In 33% of the patients, CNS involvement was part of disseminated NTM disease. Cavitary/nodular lung disease, endocarditis, cutaneous disease, lymphadenitis, bone-marrow infiltration, and osteomyelitis were other manifestations in these patients. NTM bacteremia was present in four cases (2 of these patients were HIV positive) [12–15]. HIV was the most common risk factor for NTM, followed by neurosurgical implant and corticosteroid use (Table 3). No risk factor for NTM was found in 25% (n = 28) of the patients.

**Table 3:** Clinical presentation, comorbidities and risk factors in patients with CNS NTM infection

Clinical features	Number of patients (*N* = 112)
Fever	66 (58.9%)
Headache/vomiting	40 (35.7%)
AMS (altered mental status)	33 (29.5%)
Focal neurological deficit	25 (22.3%)
Seizure	15 (13.4%)
Immunocompromised conditions/Comorbidities/Risk factors	
HIV	38 (33.9%)
Neurosurgical hardware	25 (22.3%)
Corticosteroids	8 (7.1%)
Malignancy	6 (5.4%)
Mastoiditis/Chronic suppurative otitis media (CSOM)	4 (3.6%)
End Stage Renal Disease (ESRD)	4 (3.6%)
Diabetes	3 (2.7%)
None	26 (23.2%)

### Laboratory diagnosis (radiological features and CSF findings)

Isolation of NTM from culture is the gold standard for establishing the diagnosis. Molecular methods are now being used frequently for diagnosis and species differentiation. In this review, 26 patients were diagnosed by PCR-based tests. Gene sequencing was used in 16 patients for species detection (16S rRNA was the most common gene sequenced, followed by rpoB and the hsp65 gene.) We have summarised all cases diagnosed with gene sequencing along with drug susceptibility results in Table 4 [16–30]. We also retrieved the neuroimaging findings (CT/MRI) in 82 patients. Single or multiple space-occupying lesion (mass, abscess or ring-enhancing lesion) was the most common radiological finding, closely followed by leptomeningeal enhancement (36.6% and 28%, respectively). Other common imaging findings are described in Table 5. Data regarding CSF analysis was available in 68 patients. CSF AFB smear was positive in only 26 (38.2%) patients. Interestingly, neutrophilic pleocytosis was present in nearly 40% of the cases.

**Table 4:** Summary of the drug resistant 16S rRNA, rpoB and hsp65 genes by type of NTM strain from reviewed papers

Authors (year)	NTM species	Type of gene sequenced	Resistance drugs	Susceptible drugs
Adekambi et al. 2006	*Mycobacterium mucogenicum* (ATCC 49650^ T^)	16S rRNA/rpoB	Minocycline, azithromycin, ciprofloxacin, rifampin	Amikacin, Imipenem, Clarithromycin
Adekambi et al. 2006	*Mycobacterium mucogenicum* (ATCC 49650^ T^)	16S rRNA/rpoB	Clarithromycin, ciprofloxacin	Amikacin, imipenem, doxycycline, cefoxitin
Salimanzadeh et al. 2014	*Mycobacterium chelonae*	rpoB	Not available	Doxycycline, clarithromycin
Salas et al. 2017	*Mycobacterium goodii*	rpoB	Trimethoprim–sulfamethoxazole	Doxycycline, amoxicillin–clavulanic acid
Greninger et al. 2015	*Mycobacterium immunogenum*	rpoB	Ciprofloxacin, doxycycline, cefoxitin, trimethoprim–sulfamethoxazole	Amikacin, clarithromycin
Leskinen et al. 2020	*Mycobacterium haemophilum*	hsp65	Not available	Not available
Moritz et al. 2017	*Mycobacterium goodii*	rpoB and 16S rRNA	Not available	Doxycycline, trimethoprim–sulfamethoxazole, linezolid, amikacin
Marie et al. 2003	*Mycobacterium fortuitum*	16S rRNA	Doxycycline, cefoxitin, rifampin	Amikacin, imipenem, clarithromycin
Giovanneze et al. 2018	*Mycobacterium abscessus*	hsp65	Not available	Not available
Buppajarntham et al. 2015	*Mycobacterium haemophilum*	16S rRNA	Not available	Not available
Uche et al. 2008	*Mycobacterium goodii*	16S rRNA	Clarithromycin	Imipenem, linezolid, trimethoprim–sulfamethoxazole, Amikacin
Kon et al. 2019	*Mycobacterium haemophilum*	16S rRNA	Not available	Not available
Phowthongkum et al. 2008	*Mycobacterium haemophilum*	16S rRNA	Not available	Not available
Colomba et al. 2012	*Mycobacterium abscessus sub. bolletii*	rpoB	Ciprofloxacin, clarithromycin, tigecycline, trimethoprim–sulfamethoxazole	Imipenem, minocycline, cefoxitin
Sariol et al. 2009	*Mycobacterium mucogenicum*	rpoB	Not available	Not available
Li et al. 2022	*Mycobacterium chelonae*	hsp65	Not available	Not available

**Table 5:** Neuroimaging and CSF findings in patients with nontuberculous mycobacterium infection

Radiological findings (CT/MRI) in patients with CNS NTM infections	Number of patients (*N* = 82)
Mass/abscess/Ring enhancing lesion (single or multiple)	30 (36.6%)
Leptomeningeal enhancement	23 (28%)
Hydrocephalus	12 (14.6%)
T2 hyperintensities	11 (13.4%)
ventriculitis	10 (12.2%)
Subdural empyema	3 (3.7%)
Haemorrhagic mass	2 (2.4%)
Cerebral thrombophlebitis	2 (2.4%)
Myelitis	1 (1.2%)
CSF biochemical values (total cases, *n* = 68)	
Protein (mg/dl)	87 (Median), (IQR, 51–200.8)
Glucose (mg/dl)	40.5 (Median), (IQR, 27–58.6)
Total Cells/µl	54 (Median), (IQR, 10–298)
Lymphocytic Pleocytosis	41 (60.3%)
Neutrophilic Pleocytosis	27 (39.7%)

### Treatment and outcome

Treatment of nontuberculous mycobacterial infection warrants long-term antimicrobial therapy. The median duration of treatment was 16 months in this study. Antimicrobial susceptibility is vital for treatment initiation, though DST (drug-susceptibility testing) was performed in only 41 patients. Macrolide resistance was seen in seven patients (17.07%). *Mycobacterium fortuitum* was the most common isolate in these seven patients (4 out of 7). The other three resistant isolates were *M. abscessus*, *M. gooddi* and *M. bolletii*. Quinolones (ciprofloxacin, levofloxacin, moxifloxacin) were found to be resistant in 8 cases (19.51%), and the most common resistant isolate was *M. abscessus* (5 out of 8). Intrathecal amikacin was used for treatment in 8 patients with *M. abscessus* CNS disease; 3 of them died during treatment, with a mortality rate of 37.5%. Corticosteroids are an integral part of tubercular meningitis management, notwithstanding that only 8 out of 112 patients with NTM CNS infection received corticosteroids; 4 of them died during treatment.

The overall mortality rate was 37.5% in CNS NTM infections. We also analysed the different predictors of mortality in CNS NTM infection. All patients were divided into survived and non-survived groups. Table 6 describes the comparison of both groups for various predictors of mortality. In univariate regression analysis, advanced age, HIV, Male gender, disseminated disease, *M. fortuitum*, and high CSF protein were the important determinants of outcome. After adjusting the covariates (age, comorbidities), male gender and HIV positivity were found to be the independent predictors of mortality (Table 6). In addition, *M. fortuitum* infection was the independent predictor of survival (adjusted OR 0.18, 95% CI 0.08–0.45, *p* value 0.012) with only a 4.1% mortality rate.

**Table 6:** Clinical predictors associated with mortality in patients with CNS NTM infection

Variable	Univariate analysis			Multivariate analysis	
	Mortality rate (%)	*p* value	Crude OR (95% CI)	*p* value	Crude OR (95% CI)
Age					
≥ 65 (years)	63.6	0.039	4.1, (1.2–17.2)	0.198	1.8, (0.32–3.2)
< 65	34.7				
Male	46.5	0.011	3.3, (1.2–7.3)	0.025	2.4, (1.2–7.9)
Female	21.9				
HIV	57.9	0.002	3.4, (1.5–7.9)	0.017	3.7, (1.8–6.1)
Non-HIV	27.4				
Disseminated NTM	50	0.044	2.4, (1.1–5.4)	0.181	1.5, (0.45–6.5)
Isolated CNS NTM	28.8				
MAC infection	47.4	0.149	1.2, (0.82–4.1)		
Non-MAC NTM	32.4				
*M. fortuitum* infection	4.1	< 0.001	0.05, (0.01–0.38)	0.012	0.18, (0.08–0.45)
Other NTM	46.6				
CSF protein					
≥ 60 mg/dl	63.3	0.011	3.8, (2.1–28.9)	0.091	2.3, (0.56–23.1)
< 60	18.2				

OR = Odds ratio, CI = Confidence interval, *p* value < 0.05 considered significant

## Discussion

Mycobacteria other than *Mycobacterium tuberculosis complex* and *Mycobacterium laprae* are defined as nontuberculous mycobacteria (NTM). Once considered a neglected disease, NTM is now acknowledged as an emerging pathogen that can cause a wide array of infections. In 1959, Runyon first classified NTM into four groups (photochromogens (SGM), Scotochromogens (SGM), nonphotochromogens (SGM), and RGM) based on their growth rate, morphology and pigmentation in the presence of light [31]. The incidence and prevalence of NTM disease are increasing with the advancement of laboratory diagnostic methods (molecular diagnosis, especially metagenomics) and the growing awareness among clinicians. The ageing population, use of immunosuppressants and multiple comorbidities are other factors attributing to increasing NTM infections [32].

According to a recent report, the incidence and prevalence rates of NTM disease were 17.9 and 33.3 per 100,000 population [33]. NTM is not considered a notifiable disease, resulting in under-reporting. Data are lacking regarding the precise incidence of CNS NTM disease. Most studies discussed pulmonary involvement, and some reported extrapulmonary manifestations, which involve skin, lymph nodes and bones [34, 35]. We performed a systematic review of all cases of NTM causing CNS disease; to our knowledge, this is one of the most extensive systematic review of CNS cases in the literature. Some reports include only rapidly growing NTM or MAC infections [7, 36]. A review by Flor et al. in 1996 included patients with NTM meningitis [7]; contrary to that, this study included cases with all sorts of CNS manifestations. Interestingly we found some rare findings such as cerebral thrombophlebitis and myelitis due to NTM. Previous reviews also included probable or doubtful cases, where a concomitant organism was isolated from CSF or lacked clinical features corresponding to the isolated organism [7]. Our review analysed only cases with clinical or biochemical evidence of meningitis with NTM identification by culture or molecular diagnosis.

This review found 112 cases of CNS NTM infection in the literature. We found MAC as the most common NTM species causing CNS disease in cases reported between 1980 and 2000. At the same time, the proportion of RGM has been increasing in the last two decades (from 23% in 1980–2000 to 65% in 2001–2022). Flor et al. [7] described MAC as the leading cause of CNS disease (62% incidence); however, MAC was isolated in only 34% of cases in the current review. The possible explanation for the decrease in MAC incidence is the introduction of highly active antiretroviral therapy in HIV patients [37], since MAC infections are commonly associated with HIV (67.5% in this systematic review). In advanced HIV disease, there is a decrease in TNF-alpha production along with defective/absent IFN Gamma receptors, which are the precipitating factors for MAC infections [37, 38]. This is further substantiated by a study by Winthrop et al., which reported the high incidence of MAC disease (50%) in patients receiving anti-TNF-α therapy [38].

Diagnosing NTM infections remains challenging due to its protean presentation and low index of suspicion. Radiological findings are non-specific and difficult to differentiate from tubercular and fungal CNS infections, such as neuroaspergillosis [39]. Brain abscess is a common finding that is single and supratentorial in most cases [40]. Culture remains the gold standard for final diagnosis [41, 42]. Metagenomic next-generation sequencing is now emerging as a rapid and effective tool for diagnosing NTM [30, 43]. The treatment of extrapulmonary NTM disease is not well-defined, particularly in CNS disease, where the management depends upon anecdotal evidence in the form of case reports or expert opinion. Antibiotic selection is largely based on the type of species and drug susceptibility test. The first-line antitubercular drugs have no activity against RGM. Amikacin, clarithromycin, imipenem and Quinolones are used in various combinations (minimum 3–4 drugs, preferably with good CNS penetration) [43, 44]. Cotrimoxazole could be an important drug in the maintenance phase due to its good CNS penetration. The optimal duration of therapy is not standardized, though a minimum of 12 months after culture conversion is a must [45]. Poor compliance, adverse events and drug resistance are the major deterrents during prolonged therapy. Especially macrolides resistance is a paramount concern; 17% of NTM isolates were found clarithromycin resistant in this review (mostly related to *M. fortuitum*). Previous reports also showed poor susceptibility of macrolides for *M. fortuitum* [46, 47]. There is a poor correlation between in-vitro susceptibility and a clinical response due to inducible macrolide resistance owing to the expression of the erythromycin ribosomal methyltransferase gene (erm 41) in RGM [48, 49]. Extended incubation (> 7 days) is required to detect this inducible macrolide resistance in RGM [50].

Corticosteroids are fundamental for tubercular meningitis treatment. However, no concrete evidence favours their use in CNS NTM infections. Theoretically, steroids can reduce inflammation and neurological sequelae. However, concern remains due to the possibility of increased dissemination of disease. The mortality rate in this review was lesser compared to preview reports (37.5% vs 77%). A high proportion of HIV and MAC coinfection could be the cause of this disparity [7]. In this review, three out of eight patients recovered with steroids [8, 12, 17]. One of these had MAC associated immune reconstitution inflammatory syndrome (IRIS) [12]. We also analyzed the various prognostic factors in CNS disease. Male gender and HIV infection were associated with increased mortality, whereas patients with *M fortuitum* had better survival than other species. Earlier literature also reported similar prognostic factors for pulmonary disease. However, a low mortality rate compared to CNS disease (12% vs 38%) [51]. This study identified high CSF protein as a risk factor for poor outcomes, similar to previous studies for tubercular meningitis [52, 53]. High CSF protein is an indicator of blood–brain barrier dysfunction and high immunogenicity. Whether using corticosteroids in such settings might be fruitful is a matter of further research.

This systematic review has a few significant limitations. Case reports had publication bias; furthermore, the retrospective nature of data and heterogenous patient population limit its general applicability. The information about drug susceptibility, drug adverse events, and long-term follow-up was also missing from the literature.

## Conclusion

Nontuberculous mycobacteria are an important, nonetheless underdiagnosed CNS infection with a significant mortality rate. In this report, we found RGM as an emerging cause of NTM CNS disease. Molecular diagnostics is imperative for species identification, drug susceptibility and optimal antibiotic therapy. The duration of antibiotic therapy and the role of corticosteroids are still unanswered that require further studies.

### Supplementary Information

**Additional file 1.** Supplemental files.

## Data Availability

The data sets used and/or analysed during the current study available from the corresponding author on reasonable request.

## Abbreviations

NTM: Non-tuberculous mycobacteria
MAC: *Mycobacterium avium* Complex
PRISMA: Preferred Reporting Items for Systematic Reviews and Meta-Analyses
JBI: Joanna Briggs Institute
RGM: Rapid-growing NTM
SGM: Slow-growing NTM
DST: Drug-susceptibility testing
IRIS: Immune reconstitution inflammatory syndrome
