# Supplementary material for: Epidemiology, clinical presentation, and predictors of outcome in nontuberculous mycobacterial central nervous system infection: a systematic review
Source: Trop Med Health. 2023 Sep 25;51:54. doi: 10.1186/s41182-023-00546-4 (PMC10518932; doi:10.1186/s41182-023-00546-4)
Supplement: Supplementary file 1 — Additional file 1. Supplemental files. [file 41182_2023_546_MOESM1_ESM.docx]

**Supplemental files**

**Supplemental 1: PRISMA checklist**

| **Section/topic** | **#** | **PRISMA Checklist item** | **Reported on page #** |
| --- | --- | --- | --- |
| **TITLE** | | |  |
| Title | 1 | Identify the report as a systematic review, meta-analysis, or both. | 1 |
| **ABSTRACT** | | |  |
| Structured summary | 2 | Provide a structured summary including, as applicable: background; objectives; data sources; study eligibility criteria, participants, and interventions; study appraisal and synthesis methods; results; limitations; conclusions and implications of key findings; systematic review registration number. | 3 |
| **INTRODUCTION** | | |  |
| Rationale | 3 | Describe the rationale for the review in the context of what is already known. | 4 |
| Objectives | 4 | Provide an explicit statement of questions being addressed with reference to participants, interventions, comparisons, outcomes, and study design (PICOS). | 4 |
| **METHODS** | | |  |
| Protocol and registration | 5 | Indicate if a review protocol exists, if and where it can be accessed (e.g., Web address), and, if available, provide registration information including registration number. | 5 |
| Eligibility criteria | 6 | Specify study characteristics (e.g., PICOS, length of follow-up) and report characteristics (e.g., years considered, language, publication status) used as criteria for eligibility, giving rationale. | 5 |
| Information sources | 7 | Describe all information sources (e.g., databases with dates of coverage, contact with study authors to identify additional studies) in the search and date last searched. | Supplement 2,4 |
| Search | 8 | Present full electronic search strategy for at least one database, including any limits used, such that it could be repeated. | Supplement 2 |
| Study selection | 9 | State the process for selecting studies (i.e., screening, eligibility, included in systematic review, and, if applicable, included in the meta-analysis). | 5 |
| Data collection process | 10 | Describe method of data extraction from reports (e.g., piloted forms, independently, in duplicate) and any processes for obtaining and confirming data from investigators. | 6 |
| Data items | 11 | List and define all variables for which data were sought (e.g., PICOS, funding sources) and any assumptions and simplifications made. | N/A |
| Risk of bias in individual studies | 12 | Describe methods used for assessing risk of bias of individual studies (including specification of whether this was done at the study or outcome level), and how this information is to be used in any data synthesis. | 5,6 |
| Summary measures | 13 | State the principal summary measures (e.g., risk ratio, difference in means). | N/A |
| Synthesis of results | 14 | Describe the methods of handling data and combining results of studies, if done, including measures of consistency (e.g., I^2^) for each meta-analysis. | N/A |
| **RESULTS** | | |  |
| Study selection | 17 | Give numbers of studies screened, assessed for eligibility, and included in the review, with reasons for exclusions at each stage, ideally with a flow diagram. | Figure 1 |
| Study characteristics | 18 | For each study, present characteristics for which data were extracted (e.g., study size, PICOS, follow-up period) and provide the citations. | 6 |
| Risk of bias within studies | 19 | Present data on risk of bias of each study and, if available, any outcome level assessment (see item 12). | Supplement 3 |
| Results of individual studies | 20 | For all outcomes considered (benefits or harms), present, for each study: (a) simple summary data for each intervention group (b) effect estimates and confidence intervals, ideally with a forest plot. | Table 1-6 |
| Synthesis of results | 21 | Present results of each meta-analysis done, including confidence intervals and measures of consistency. | N/A |
| Risk of bias across studies | 22 | Present results of any assessment of risk of bias across studies (see Item 15). | N/A |
| Additional analysis | 23 | Give results of additional analyses, if done (e.g., sensitivity or subgroup analyses, meta-regression [see Item 16]). | N/A |
| **DISCUSSION** | | |  |
| Summary of evidence | 24 | Summarize the main findings including the strength of evidence for each main outcome; consider their relevance to key groups (e.g., healthcare providers, users, and policy makers). | 9-12 |
| Limitations | 25 | Discuss limitations at study and outcome level (e.g., risk of bias), and at review-level (e.g., incomplete retrieval of identified research, reporting bias). | 12 |
| Conclusions | 26 | Provide a general interpretation of the results in the context of other evidence, and implications for future research. | 12 |
| **FUNDING** | | |  |
| Funding | 27 | Describe sources of funding for the systematic review and other support (e.g., supply of data); role of funders for the systematic review. | 12 |

**Supplemental 2**

Search Strategy Report

**PubMed (275 documents)**

**(((((Central nervous system) OR (CNS)) OR (Brain abscess)) OR (Meningitis)) OR (spinal)) AND ((NTM) OR (Nontuberculous mycobacteria))**

("1980/01/01"[PubDate] : "2022/12/31"[PubDate])

**Google Scholar (380 documents)**

central nervous system CNS NTM nontuberculous mycobacterium meningitis brain abscess

**Scopus (195 documents)**

( TITLE-ABS-KEY ( central  AND nervous  AND system )  OR  TITLE-ABS-KEY ( cns )  OR  TITLE-ABS-KEY ( brain  AND abscess )  OR  TITLE-ABS-KEY ( meningitis )  OR  TITLE-ABS-KEY ( spinal )  AND  TITLE-ABS-KEY ( nontuberculous  AND mycobacteria )  OR  TITLE-ABS-KEY ( ntm ) )

**Supplemental 3: Quality assessment**

Table1: JBI Critical Appraisal Checklist for Case Reports

| Study reference | Were patient’s demographic characteristics clearly described? | Was the patient’s history clearly described and presented as a timeline?? | Was the current clinical condition of the patient on presentation clearly described? | Were diagnostic tests or methods and the results clearly described? | Was the intervention(s) or treatment procedure(s) clearly described? | Was the post-intervention clinical condition clearly described? | Were adverse events (harms) or unanticipated events identified and described?? | Does the case report provide takeaway lessons? | Risk of bias^a^ |
| --- | --- | --- | --- | --- | --- | --- | --- | --- | --- |
| Zakowski et al 1982 | yes | yes | yes | yes | yes | yes | yes | yes | low |
| d'Incau et al 2020 | yes | yes | no | yes | yes | yes | no | yes | moderate |
| Chowdhary et al 2015 | yes | yes | no | yes | yes | yes | no | yes | moderate |
| Karne et al 2012 | yes | yes | yes | yes | yes | yes | yes | yes | low |
| Arkun et al 2012 | yes | yes | yes | yes | yes | yes | no | yes | low |
| Begley et al 2014 | yes | yes | yes | yes | yes | yes | yes | yes | low |
| Xess et al 2019 | yes | yes | yes | yes | yes | yes | yes | yes | low |
| Morrison et al 1999 | yes | yes | yes | yes | yes | yes | yes | yes | low |
| Uldry et al 1992 | yes | yes | no | yes | yes | yes | no | no | moderate |
| Verma et al 2009 | yes | yes | yes | no | yes | yes | no | yes | low |
| Burger et al 2004 | yes | yes | yes | yes | yes | yes | no | yes | low |
| Murray et al 2001 | yes | yes | no | yes | yes | yes | yes | yes | low |
| Montero et al 2016 | yes | yes | no | yes | yes | yes | yes | yes | low |
| Viswanathan et al 2004 | yes | yes | no | yes | yes | yes | yes | yes | low |
| Baidya et al 2016 | yes | yes | yes | yes | yes | yes | yes | yes | low |
| Aliabadi et al 2008 | yes | yes | yes | yes | yes | no | yes | yes | low |
| Leskinen et al 2020 | yes | yes | yes | yes | no | yes | yes | yes | low |
| Sardar et al 2009 | yes | yes | no | no | yes | yes | no | yes | moderate |
| Maniu et al 2001 | yes | yes | no | no | yes | yes | no | yes | moderate |
| Quinn et al 2002 | yes | yes | no | no | yes | yes | no | yes | moderate |
| Dalovisio et al 1981 | yes | yes | no | no | yes | no | no | yes | high |
| Midani et al 1999 | yes | yes | no | no | yes | yes | no | yes | moderate |
| Talati et al 2008 | yes | yes | yes | yes | no | yes | yes | yes | low |
| Moritz et al 2017 | yes | yes | no | yes | yes | yes | yes | yes | low |
| Little et al 2006 | yes | yes | no | yes | yes | no | yes | yes | moderate |
| Madaras-Kelly et al 1999 | yes | yes | no | no | yes | yes | yes | yes | moderate |
| Liebeskind et al 2001 | yes | yes | yes | yes | yes | yes | yes | yes | low |
| Marie et al 2003 | yes | yes | no | yes | yes | yes | no | yes | low |
| Levy et al 2016 | yes | yes | no | no | yes | yes | no | yes | moderate |
| Damiano et al 2018 | yes | yes | no | yes | yes | yes | yes | yes | low |
| Buppajarntham et al 2015 | yes | yes | yes | yes | yes | yes | yes | yes | low |
| Cai et al 2014 | yes | yes | no | yes | yes | yes | no | yes | moderate |
| Chan et al 1991 | yes | yes | yes | yes | yes | yes | yes | yes | low |
| Lee et al. 2012 | yes | yes | no | yes | no | yes | no | yes | moderate |
| Padmanaban et al 2020 | yes | yes | yes | yes | yes | yes | no | yes | low |
| Zakrzewski et al 2019 | yes | yes | yes | yes | yes | no | yes | yes | low |
| Cadena et al 2014 | yes | yes | no | yes | yes | yes | no | yes | low |
| Uche et al 2008 | yes | yes | no | yes | yes | yes | no | yes | low |
| Know et al 2018 | yes | yes | no | yes | yes | yes | no | yes | low |
| Flor et al 1996 | yes | yes | yes | yes | yes | yes | no | yes | low |
| Mankad et al 2015 | yes | yes | yes | yes | yes | yes | yes | yes | low |
| Adekambi et al 2006 | yes | yes | no | no | no | yes | yes | yes | moderate |
| Salmanzadeh et al 2014 | yes | yes | yes | yes | yes | yes | no | yes | low |
| Greninger et al 2015 | yes | yes | yes | yes | yes | yes | yes | yes | low |
| Salas et al 2017 | yes | yes | no | no | yes | yes | no | yes | moderate |
| Tankhiwale et al 2014 | yes | yes | no | yes | no | yes | no | yes | moderate |
| Kumar et al 1989 | yes | yes | no | no | no | yes | no | yes | high |
| Lee et al 2013 | yes | yes | yes | yes | yes | no | yes | yes | low |
| Onor et al 2017 | yes | yes | yes | yes | yes | yes | yes | yes | low |
| Kon et al 2019 | yes | yes | yes | yes | yes | yes | no | yes | low |
| Phowthongkum et al 2008 | yes | yes | yes | yes | yes | yes | yes | yes | low |
| Wallace et al 1983 | yes | yes | no | yes | yes | no | no | no | high |
| Smith et al 1996 | yes | yes | yes | yes | yes | yes | yes | yes | low |
| Fujikawa et al 2006 | yes | yes | yes | yes | yes | yes | yes | yes | low |
| Jacob et al 1993 | yes | no | no | yes | no | no | yes | yes | high |
| Santamaría-Jaúregui et al 1984 | yes | no | yes | no | no | yes | yes | yes | moderate |
| Moiz et al 2020 | yes | yes | yes | yes | yes | yes | yes | yes | low |
| J.Clay Goodman et al 2020 | yes | yes | yes | no | no | yes | no | no | high |
| Lamb et al 2019 | yes | yes | yes | yes | yes | yes | yes | yes | low |
| Tondon et al 2007 | yes | yes | yes | yes | yes | yes | yes | yes | low |
| Smith et al 2003 | yes | yes | yes | yes | yes | yes | yes | yes | low |
| Suttner et al 2001 | yes | yes | yes | yes | yes | yes | yes | yes | low |
| Dash et al 2020 | yes | yes | no | yes | yes | yes | no | yes | moderate |
| Nookeu et al 2019 | yes | yes | yes | yes | yes | yes | yes | yes | low |
| Colomba et al 2012 | yes | yes | yes | yes | yes | no | no | yes | moderate |
| Sariol et al 2009 | yes | yes | yes | yes | yes | no | yes | yes | low |
| Wu EL et al 2022 | yes | yes | yes | yes | no | yes | yes | yes | low |
| Choi et al 2021 | yes | yes | yes | yes | no | yes | no | yes | moderate |
| Clabots et al 2022 | yes | yes | yes | yes | yes | yes | yes | no | low |
| Pacholec et al 2020 | yes | yes | yes | yes | no | yes | no | yes | moderate |
| Li Y et al 2022 | yes | yes | yes | yes | no | yes | yes | yes | low |
| Lane-Donovan et al 2021 | yes | yes | yes | no | yes | yes | yes | yes | low |
| Gupta et al 2020 | yes | yes | no | yes | yes | no | no | yes | moderate |
| Abu-ghaname et al 2020 | yes | yes | yes | yes | yes | yes | no | yes | low |
| Rotter et al 2020 | yes | yes | yes | yes | yes | yes | no | yes | low |
| Lim et al 2016 | yes | yes | yes | no | yes | no | yes | yes | moderate |

^a^ Risk based on eight questions (Low risk = ≥ 7 yes; Moderate risk = 5-6 yes; High risk = ≤ 4 yes).

**Supplemental-4 (All references included in this review)**

1. Zakowski P, Fligiel S, Berlin GW, Johnson L Jr. Disseminated Mycobacterium avium-intracellulare infection in homosexual men dying of acquired immunodeficiency. JAMA 1982;248:2980-2
2. d'Incau S, Vargas MI, Calmy A, Janssens JP. *Mycobaterium fortuitum* disseminated infection in an immunocompetent patient without predisposing factors. BMJ Case Rep. 2020 Sep 29;13(9):e235842.
3. Chowdhary M, Narsinghani U, Kumar RA. Intracranial abscess due to Mycobacterium avium complex in an immunocompetent host: a case report. BMC Infect Dis. 2015 Jul 23;15:281. doi: 10.1186/s12879-015-1026-5.
4. Karne SS, Sangle SA, Kiyawat DS, Dharmashale SN, Kadam DB, Bhardwaj RS. Mycobacterium avium-intracellulare brain abscess in HIV-positive patient. Ann Indian Acad Neurol. 2012 Jan;15(1):54-5.
5. Arkun K, Gordon DA, Lincoln C, Levi M, Bello J, Keller CE, Weidenheim KM. Atypical mycobacterial brain abscess presenting as a spindle cell lesion in an immunocompetent patient. Clin Neuropathol. 2012 May-Jun;31(3):155-8.
6. Begley C, Amaraneni A, Lutwick L. Mycobacterium avium-intracellulare brain abscesses in an HIV-infected patient. IDCases. 2014 Nov 24;2(1):19-21.
7. Xess AB, Bala K, Panigrahy A, Singh U. *Mycobacterium fortuitum* as a cause of acute CNS infection in an immune-competent girl undergoing repeated VP shunt surgeries. BMJ Case Rep. 2019 Apr 15;12(4):e226900.
8. Morrison A, Gyure KA, Stone J, Wong K, McEvoy P, Koeller K, Mena H. Mycobacterial spindle cell pseudotumor of the brain: a case report and review of the literature. Am J Surg Pathol. 1999 Oct;23(10):1294-9.
9. Uldry PA, Bogousslavsky J, Regli F, Chave JP, Beer V. Chronic Mycobacterium avium complex infection of the central nervous system in a nonimmunosuppressed woman. Eur Neurol. 1992;32(5):285-8.
10. Verma R, Dhamija R. Disseminated Mycobacterium avium-intracellulare infection presenting as multiple ring-enhancing lesions on brain MRI. Mayo Clin Proc. 2009 May;84(5):394.
11. Berger P, Lepidi H, Drogoul-Vey MP, Poizot-Martin I, Drancourt M. Mycobacterium avium brain abscess at the initiation of highly active antiretroviral therapy. Eur J Clin Microbiol Infect Dis. 2004 Feb;23(2):142-4.
12. Murray R, Mallal S, Heath C, French M. Cerebral mycobacterium avium infection in an HIV-infected patient following immune reconstitution and cessation of therapy for disseminated mycobacterium avium complex infection. Eur J Clin Microbiol Infect Dis. 2001 Mar;20(3):199-201
13. Montero JA, Alrabaa SF, Wills TS. Mycobacterium abscessus ventriculoperitoneal shunt infection and review of the literature. Infection. 2016 Apr;44(2):251-3.
14. Viswanathan R, Bhagwati SN, Iyer V, Newalkar P. Ventriculo-peritoneal shunt infection by mycobacterium fortuitum in an adult. Neurol India. 2004 Sep;52(3):393-4.
15. Baidya A, Tripathi M, Pandey P, Singh UB. Mycobacterium abscessus as a Cause of Chronic Meningitis: A Rare Clinical Entity. Am J Med Sci. 2016 Apr;351(4):437-9.
16. Aliabadi H, Osenbach RK. Intrathecal Drug Delivery Device Infection and Meningitis due to Mycobacterium Fortuitum: A Case Report. Neuromodulation. 2008 Oct;11(4):311-4
17. Leskinen, S., Flowers, X., Thoene, K. *et al.* Meningomyeloencephalitis secondary to *Mycobacterium haemophilum* infection in AIDS. *acta neuropathol commun* **8,**73 (2020).
18. Sardar P, Bandyopadhyay D, Roy D, Guha P, Guha G, Banerjee AK. Non Tuberculous Mycobacteria and toxoplasma co-infection of the central nervous system in a patient with AIDS. Braz J Infect Dis. 2009 Dec;13(6):449-51.
19. Maniu CV, Hellinger WC, Chu SY, Palmer R, Alvarez-Elcoro S. Failure of treatment for chronic Mycobacterium abscessus meningitis despite adequate clarithromycin levels in cerebrospinal fluid. Clin Infect Dis. 2001 Sep 1;33(5):745-8.
20. Quinn JV, Steele RW. Ventriculitis following a neurosurgic procedure. Clin Pediatr (Phila). 2002 Jul-Aug;41(6):439-41.
21. Dalovisio JR, Pankey GA, Wallace RJ, Jones DB. Clinical usefulness of amikacin and doxycycline in the treatment of infection due to Mycobacterium fortuitum and Mycobacterium chelonei. Rev Infect Dis. 1981 Sep-Oct;3(5):1068-74.
22. Midani S, Rathore MH. Mycobacterium fortuitum infection of ventriculoperitoneal shunt. South Med J. 1999 Jul;92(7):705-7. doi: 10.1097/00007611-199907000-00009.
23. Talati NJ, Rouphael N, Kuppalli K, Franco-Paredes C. Spectrum of CNS disease caused by rapidly growing mycobacteria. Lancet Infect Dis. 2008 Jun;8(6):390-8.
24. Moritz DC, Harrington AT, Slavin K, Gomez C, Jarrett OD. Deep brain stimulator infection by a novel rapid growing mycobacterium. Acta Neurochir (Wien). 2017 Nov;159(11):2239-2241.
25. Little AA, Gebarski SS, Blaivas M. Nontuberculous mycobacterial infection of a metastatic brain neoplasm in an immunocompromised patient. Arch Neurol. 2006 May;63(5):763-5.
26. Madaras-Kelly KJ, DeMasters TA, Stevens DL. Mycobacterium fortuitum meningitis associated with an epidural catheter: case report and a review of the literature. Pharmacotherapy. 1999 May;19(5):661-6.
27. Liebeskind DS, Ostrzega N, Wasterlain CG, Buttner EA. Neurologic manifestations of disseminated infection with Mycobacterium abscessus. Neurology. 2001 Mar 27;56(6):810-3.
28. Marie I, Héron F, Lecomte F, Jarlier V, Truffot-Pernot C, Laquerriere A, Huerre M, Levesque H, Courtois H. Multiple cerebral abscesses as a complication of Mycobacterium fortuitum infection. Eur J Intern Med. 2003 Oct;14(6):386-389.
29. Levy ZD, Du V, Chiluwal A, Chalif DJ, Ledoux DE. Ventriculoperitoneal Shunt Infection with Mycobacterium abscessus: A Rare Cause of Ventriculitis. World Neurosurg. 2016 Feb;86:510.e1-4.
30. Giovannenze F, Stifano V, Scoppettuolo G, Damiano F, Pallavicini F, Delogu G, Palucci I, Rapisarda A, Sturdà C, Pompucci A. Incidental intraoperative diagnosis of Mycobacterium abscessus meningeal infection: a case report and review of the literature. Infection. 2018 Oct;46(5):591-597.
31. Buppajarntham A, Apisarnthanarak A, Rutjanawech S, Khawcharoenporn T. Central nervous system infection due to Mycobacterium haemophilum in a patient with acquired immunodeficiency syndrome. Int J STD AIDS. 2015 Mar;26(4):288-90.
32. Cai R, Qi T, Lu H. Central nervous system infection with non-tuberculous mycobacteria: a report of that infection in two patients with AIDS. Drug Discov Ther. 2014 Dec;8(6):276-9.
33. Chan KH, Mann KS, Seto WH. Infection of a shunt by Mycobacterium fortuitum: case report. Neurosurgery. 1991 Sep;29(3):472-4.
34. Lee MR, Cheng A, Lee YC, Yang CY, Lai CC, Huang YT, Ho CC, Wang HC, Yu CJ, Hsueh PR. CNS infections caused by Mycobacterium abscessus complex: clinical features and antimicrobial susceptibilities of isolates. J Antimicrob Chemother. 2012 Jan;67(1):222-5.
35. Padmanaban V, Hussein R, Rizk E. Nontuberculous Mycobacterial Infection in Patients with Neurosurgical Hardware: Two Cases and A Review of the Literature. Cureus. 2020 Mar 24;12(3):e7398.
36. Zakrzewski J, Hu K, Neisewander BL, Esfahani DR, Bhimani AD, Shah HP, Haddadin DW, Mehta AI. *Mycobacterium fortuitum* Meningitis: Approach to Lumboperitoneal Shunt Infection. South Med J. 2019 Apr;112(4):217-221.
37. Cadena G, Wiedeman J, Boggan JE. Ventriculoperitoneal shunt infection with Mycobacterium fortuitum: a rare offending organism. J Neurosurg Pediatr. 2014 Dec;14(6):704-7.
38. Uche CS, Silibovsky R, Jungkind D, Measley R. Ventriculoperitoneal shunt-associated Mycobacterium goodii infection. Infect Dis Clin Pract. 2008;16:129–130.
39. Kwon LM, Kim ES, Lee K, Lee Y, Song JH (2018) young healthy adult: A case report and literature review. Radiol Infect Dis 5: 85–90.
40. Flor A, Capdevila JA, Martin N, Gavaldà J, Pahissa A. Nontuberculous mycobacterial meningitis: report of two cases and review. Clin Infect Dis. 1996 Dec;23(6):1266-73.
41. Mankad S, Karthik R, Rupali P, Michael JS. Fatal Disseminated Mycobacterium chelonae Infection in an Immunocompromised Host--A Unique Presentation. J Assoc Physicians India. 2015 Jan;63(1):49-52.
42. Adékambi T, Foucault C, La Scola B, Drancourt M. Report of two fatal cases of Mycobacterium mucogenicum central nervous system infection in immunocompetent patients. J Clin Microbiol. 2006 Mar;44(3):837-40.
43. Salmanzadeh S, Honarvar N, Goodarzi H, Khosravi AD, Nashibi R, Serajian AA, Hashemzadeh M. Chronic mycobacterial meningitis due to Mycobacterium chelonae: a case report. Int J Infect Dis. 2014 Oct;27:67-9.
44. Greninger AL, Langelier C, Cunningham G, Keh C, Melgar M, Chiu CY, Miller S. Two Rapidly Growing Mycobacterial Species Isolated from a Brain Abscess: First Whole-Genome Sequences of Mycobacterium immunogenum and Mycobacterium llatzerense. J Clin Microbiol. 2015 Jul;53(7):2374-7.
45. Salas NM, Klein N. *Mycobacterium goodii:* An Emerging Nosocomial Pathogen: A Case Report and Review of the Literature. Infect Dis Clin Pract (Baltim Md). 2017 Mar;25(2):62-65.
46. Tankhiwale SS, Katkar VJ. Subdural empyma due to Mycobacterium fortuitum in a non-HIV patient. Indian J Med Microbiol. 2014 Oct-Dec;32(4):446-8.
47. Kumar L, Shanta V. Meningitis due to Mycobacterium kansasi in nonHodgkin's lymphoma. N Z Med J. 1989 Sep 27;102(876):516.
48. Lee YC, Lu CL, Lai CC, Tseng YT, Sun HY, Hung CC. Mycobacterium avium complex infection-related immune reconstitution inflammatory syndrome of the central nervous system in an HIV-infected patient: case report and review. J Microbiol Immunol Infect. 2013 Feb;46(1):68-72.
49. Onor IO, Piazza ME, Khashan MF, Walvekar S, Guillory SG. Mycobacterium kansasii: A Rare Cause of Brain Abscess. Am J Med Sci. 2017 Jan;353(1):90-92.
50. Kon S, Franco-Paredes C, Hawkins KL. Intramedullary spinal cord lesions in an immunocompromised host due to *Mycobacterium haemophilum*. IDCases. 2019 Nov 22;19:e00674.
51. Phowthongkum P, Puengchitprapai A, Udomsantisook N, Tumwasorn S, Suankratay C. Spindle cell pseudotumor of the brain associated with Mycobacterium haemophilum and Mycobacterium simiae mixed infection in a patient with AIDS: the first case report. Int J Infect Dis. 2008 Jul;12(4):421-4.
52. Wallace RJ Jr, Swenson JM, Silcox VA, Good RC, Tschen JA, Stone MS. Spectrum of disease due to rapidly growing mycobacteria. Rev Infect Dis. 1983 Jul-Aug;5(4):657-79.
53. Smith MB, Boyars MC, Woods GL. Fatal Mycobacterium fortuitum meningitis in a patient with AIDS. Clin Infect Dis. 1996 Dec;23(6):1327-8.
54. Fujikawa K, Suenaga A, Motomura M, Fukuda T, Ooe N, Eguchi K. [A case of Mycobacterium fortuitum meningitis following surgery for meningioma]. Rinsho Shinkeigaku. 2006 Jul;46(7):480-4.
55. Jacob CN, Henein SS, Heurich AE, Kamholz S. Nontuberculous mycobacterial infection of the central nervous system in patients with AIDS. South Med J. 1993 Jun;86(6):638-40.
56. Santamaría-Jaúregui J, Sanz-Hospital J, Berenguer J, Muñoz D, Gómez-Mampaso E, Bouza E. Meningitis caused by Mycobacterium fortuitum. Am Rev Respir Dis. 1984 Jul;130(1):136-7.
57. Moiz S, Rahman O, Morcos M, Siddiqui A, Hameed UB. Mycobacterium mucogenicum meningitis due to external ventricular drain. Access Microbiol. 2020 acmi000167.
58. J.Clay Goodman, Shankar Gopinath, Wedad Alfarkh, Kenneth Muldrew, Andrew DiNardo Mycobacteria avium complex (MAC) Brain Abscess in an AIDS Patient: Case Report and Literature Review (4993). Neurology Apr 2020, 94 (15 Supplement) 4993;
59. Lamb GS, Del Valle Mojica C, Srinivas N, Starke J. Central Nervous System Infections Caused by Mycobacterium abscessus: Ventricular Shunt Infection in Two Pediatric Patients and Literature Review. Pediatr Infect Dis J. 2019 May;38(5):e99-e104.
60. R. Tandon, K. S. Kim, and R. Serrao, “Disseminated *Mycobacterium avium-intracellulare* infection in a person with AIDS with cutaneous and CNS lesions,” *AIDS Reader*, vol. 17, no. 11, pp. 555–560, 2007.
61. Smith MB, Molina CP, Schnadig VJ, Boyars MC, Aronson JF. Pathologic features of Mycobacterium kansasii infection in patients with acquired immunodeficiency syndrome. Arch Pathol Lab Med. 2003 May;127(5):554-60.
62. Suttner NJ, Adhami Z, Aspoas AR. Mycobacterium chelonae lumbar spinal infection. Br J Neurosurg. 2001 Jun;15(3):265-9.
63. Dash A, Gupta N, Ray Y, Kodan P, Singh BK, Soneja M. Choosing the therapy for neurological infection with rapidly growing mycobacteria. Drug Discov Ther. 2020 Sep 8;14(4):211-212.
64. Nookeu P, Angkasekwinai N, Foongladda S, Phoompoung P. Clinical Characteristics and Treatment Outcomes for Patients Infected with Mycobacterium haemophilum. Emerg Infect Dis. 2019;25(9):1648-1652.
65. Colomba C, Rubino R, Di Carlo P, Mammina C, Bonura C, Siracusa L, Titone L, Saporito L. Probable disseminated Mycobacterium abscessus subspecies bolletii infection in a patient with idiopathic CD4+ T lymphocytopenia: a case report. J Med Case Rep. 2012 Sep 4;6:277. doi: 10.1186/1752-1947-6-277. PMID: 22947084; PMCID: PMC3514384.
66. Sariol CA, Galib Y, Pantoja P, Colón L, González A, Tormos LM, Santana J, Luciano CA, González-Martínez J, Kraiselburd EN. Fatal granulomatous meningoencephalitis associated to mycobacterium mucogenicum-like microorganism: a case report. P R Health Sci J. 2009 Sep;28(3):276-80. PMID: 19715122; PMCID: PMC2909603.
67. Wu EL, Al-Heeti O, Hoff BM, Williams JL, Krueger KM, Santoiemma PP, Rhodes NJ. Role of Therapeutic Drug Monitoring in the Treatment of Persistent *Mycobacterium abscessus* Central Nervous System Infection: A Case Report and Review of the Literature. Open Forum Infect Dis. 2022 Jul 30;9(8):ofac392. doi: 10.1093/ofid/ofac392
68. Wang L, Wang F, Yang C, Luo F. Central nervous system infection caused by *Mycobacterium houstonense*: A case report. Front Neurol. 2022 Sep 1;13:908086. doi: 10.3389/fneur.2022.908086.
69. Choi et al. Disseminated Mycobacterium avium Complex Infection Causing Multiple Skull Defects in an Immunocompetent Patient: A Case ReportJ Wound Manag Res. 2021;17 (3): 202-206.
70. Clabots D, Serrat A. *Mycobacterium abscessus* peritonitis and ventriculitis associated with ventriculoperitoneal shunt. IDCases. 2022 Feb 11;27:e01445. doi: 10.1016/j.idcr.2022.e01445.
71. Pacholec M, Sami F, Newell K, El Atrouni W. Fatal disseminated *Mycobacterium haemophilum* infection involving the central nervous system in a renal transplant recipient. J Clin Tuberc Other Mycobact Dis. 2020 Oct 19;21:100197. doi: 10.1016/j.jctube.2020.100197.
72. Li Y, Zhang L, Li C, Zou X, Zeng Y, Hu Z, Xiao B, Long L. A rare case of Mycobacterium Chelonae infection in an immunocompromised adult with cavernous sinus syndrome. CNS Neurosci Ther. 2022 May;28(5):796-799. doi: 10.1111/cns.13808.
73. Lane-Donovan C, Bainbridge E, Szumowski J, Kerkhoff AD, Peluso MJ. *Mycobacterium avium*Complex Infection as a Rare Cause of Cerebral Mass Lesion and IRIS in a Patient With AIDS: Case Report and Review of the Literature. Open Forum Infect Dis. 2021 Oct 30;8(11):ofab450. doi: 10.1093/ofid/ofab450.
74. Gupta N, Mittal A, Niyas VKM, Banerjee S, Ray Y, Kodan P, Malla S, Khot W, Fazal F, Singh BK, Jorwal P, Nischal N, Soneja M, Wig N. Nontuberculous mycobacteria: A report of eighteen cases from a tertiary care center in India. Lung India. 2020 Nov-Dec;37(6):495-500.
75. Abu-Ghname A, Davis MJ, Davies LW, Whitehead WE, Buchanan EP. Pediatric Ventriculoperitoneal Shunt Infection: The Role of Shunt Tract Debridement in Mycobacterium abscessus Eradication. J Craniofac Surg. 2020 Jan/Feb;31(1):278-282.
76. Rotter J, Graffeo CS, Perry A, Gilder HE, Wilson JW, Link MJ. Polymicrobial Intracerebral Abscess Growing Mycobacterium avium Complex and Achromobacter xylosoxidans: Case Report and Literature Review. World Neurosurg. 2020 Sep;141:441-447.e1.
77. Lim MS, Bermingham N, O'Broin C, Khalil A, Keohane C, Lim C. Isolated Cerebellar Spindle Cell Pseudotumor Caused by Mycobacterium Avium-Intracellulare Complex in a Patient without AIDS. World Neurosurg. 2016 Jun;90:703.e1-703.e3.
